# Dosimetric effects of anatomical deformations and positioning errors in VMAT breast radiotherapy

**DOI:** 10.1002/acm2.12409

**Published:** 2018-07-05

**Authors:** Maija Rossi, Eeva Boman, Tanja Skyttä, Mikko Haltamo, Marko Laaksomaa, Mika Kapanen

**Affiliations:** ^1^ Medical Imaging Centre Department of Physics Tampere University Hospital Tampere Finland; ^2^ Department of Oncology Tampere University Hospital Tampere Finland

**Keywords:** breast cancer, CBCT, tissue deformation, VMAT

## Abstract

**Aim:**

Traditional radiotherapy treatment techniques of the breast are insensitive for deformations and swelling of the soft tissue. The purpose of this study was to evaluate the dose changes seen with tissue deformations using different image matching methods when VMAT technique was used, and compare these with tangential technique.

**Methods:**

The study included 24 patients with breast or chest wall irradiations, nine of whom were bilateral. In addition to planar kV setup imaging, patients underwent weekly cone‐beam computed tomography (CBCT) imaging to evaluate soft tissue deformations. The effect of the deformations was evaluated on VMAT plans optimized with 5‐mm virtual bolus to create skin flash, and compared to standard tangential plans with 2.5 cm skin flash. Isocenter positioning using 2D imaging and CBCT were compared.

**Results:**

With postural changes and soft tissue deformations, the target coverage decreased more in the VMAT plans than in the tangential plans. The planned V90% coverage was 98.3% and 99.0% in the tangential and VMAT plans, respectively. When tattoo‐based setup and online 2D match were used, the coverage decreased to 97.9% in tangential and 96.5% in VMAT plans (*P* < 0.001). With automatic CBCT‐based image match the respective coverages were 98.3% and 98.8%. In the cases of large soft tissue deformations, the replanning was needed for the VMAT plan, whereas the tangential plan still covered the whole target volume.

**Conclusions:**

The skin flash created using an optimization bolus for VMAT plans was in most cases enough to take into account the soft tissue deformations seen in breast VMAT treatments. However, in some cases larger skin flash or replanning were needed. The use of 2D match decreased the target coverage for VMAT plans but not for FinF plans when compared to 3D match. The use of CBCT match is recommended when treating breast/chest wall patients with VMAT technique.

## 1 INTRODUCTION

Adjuvant radiotherapy (RT) is recommended for breast cancer patients after breast‐conserving surgery or node‐positive (N+) mastectomy.^1^ For a variety of treatment sites, such as prostate, brain, or head and neck, volumetric‐modulated arc therapy (VMAT) treatment technique has been implemented rapidly into practice.^2^ However, the RT of the breast is still largely accomplished with tangential field technique; not only due to the sparing of the contralateral breast from small doses caused by beam tails but also due to the possibility to account for possible swelling or deformation of the breast tissue during the treatment course using large field spillage outside the skin contour.

The VMAT has been shown to be a feasible treatment option for adjuvant RT of the breast.^3^, ^4^, ^5^, ^6^, ^7^, ^8^, ^9^ It has been shown to reduce the dose to the ipsilateral lung for both left‐ and right‐sided treatments, and to the heart for left‐sided targets.^5^, ^6^ However, in VMAT the allowance for tissue deformations is limited. In the Monaco treatment planning system (Elekta AB, Stockholm, Sweden), the skin flash tool is implemented inside the VMAT plan optimizer.^5^ In tomotherapy (TomoTherapy Hi‐Art system, Madison, USA), the optimization of the dose to the skin is possible, but to allow for swelling, further extension of the field fluences requires additional bolus structures.^10^ In the Eclipse treatment planning system (Varian Medical Systems, Palo Alto, CA, USA), there is currently no built‐in tool to account for skin spillage in the VMAT planning and some clinics use a virtual bolus on the skin surface to create the skin flash.^4^, ^6^ The virtual bolus (0.5–1.0 cm thick) is only used in the VMAT optimization phase at locations where the planning target volume (PTV) is contoured to reach the skin surface with sufficient margin, and the bolus is removed for the final dose calculation.^4^, ^6^

The purpose of this study was to investigate the dosimetric effects of swelling, shrinking or other deformation of the breast or chest wall present in the RT treatment. The deformation was determined from weekly cone‐beam computed tomography (CBCT) images collected from the breast VMAT treatments. The dosimetric effects of the deformations were investigated on the planning target volume (PTV) and heart dose parameters for different patient setups.

## 2 MATERIALS AND METHODS

### 2.A Patients

The inclusion criteria to this study were women being treated for breast cancer with adjuvant RT, and the breast or chest wall being treated with VMAT technique. In our clinic, patients are treated with VMAT if the dose constrains to the lung or heart are not met with sufficient PTV coverage using tangential half‐blocked fields with the field in field technique (FinF). The planning criteria are less than 30% of the ipsilateral lung volume receiving 20 Gy; the mean dose to the ipsilateral lung being less than 15 Gy; and the mean dose to the heart being less than 3–5 Gy. Additionally, all bilateral breast or chest wall treatments are treated with VMAT due to better dose conformity.

Twenty‐four patients were included in the study, as described in Table 1. Nine patients had bilateral breast treatment, resulting in a total of 33 PTVs. The PTVs were drawn with 5 mm margin to the clinical target volume. The axillary lymph nodes were included in all patients except for bilateral cases Pat #19 and #22 on the right side; and Pat #11 bilaterally. In 16 patients, deep inspiration breath hold (DIBH) technique was used (RPM, Varian Medical systems) to decrease the dose to the heart and lung. All patients were treated to the dose of 50 Gy in 2‐Gy daily fractions. In addition, two patients (Pat #15 and Pat #22) received a simultaneously integrated boost (SIB) to 56.25 Gy in 2.25‐Gy daily fractions.

**Table 1:** Patient characteristics describing age, side of treatment, type of surgery, and breathing technique

	Age	Treatment side	Type	Breathing technique
Pat #1	73	Right	Mastectomy	FB
Pat #2	68	Left	Mastectomy	DIBH
Pat #3	75	Left	Mastectomy	FB
Pat #4	58	Right	Mastectomy	DIBH
Pat #5	85	Left	Mastectomy	FB
Pat #6	73	Left	Conserving surgery	FB
Pat #7	39	Left	Mastectomy	DIBH
Pat #8	82	Bilateral	Mastectomy	FB
Pat #9	72	Bilateral	Mastectomy	FB
Pat #10	52	Right	Mastectomy	DIBH
Pat #11	69	Bilateral	Conserving surgery	DIBH
Pat #12	69	Left	Conserving surgery	FB
Pat #13	49	Left	Mastectomy	DIBH
Pat #14	53	Left	Mastectomy	DIBH
Pat #15^a^	58	Right	Mastectomy	DIBH
Pat #16	49	Bilateral	Conserving surgery	DIBH
Pat #17	72	Right	Conserving surgery	FB
Pat #18	49	Bilateral	Mastectomy	DIBH
Pat #19	50	Bilateral	Conserving surgery	DIBH
Pat #20	54	Bilateral	Mastectomy	DIBH
Pat #21	49	Left	Conserving surgery	DIBH
Pat #22^a^	47	Bilateral	Conserving surgery	DIBH
Pat #23	49	Bilateral	Mastectomy	DIBH
Pat #24	65	Left	Mastectomy	DIBH

FB, free breathing; DIBH, deep inspiration breath hold.

a Patient had simultaneously integrated boost to 56.25 Gy in 2.25‐Gy fractions.

### 2.B Treatment planning

The patients were imaged with computed tomography (CT) using 3‐mm slice thickness (Philips Brilliance Big Bore, Philips Medical Systems, Eindhoven, The Netherlands; or Toshiba Aquilion LB, Canon Medical Systems, Ōtawara, Japan). Patient immobilization (supine) was achieved using Candor's ConBine (Candor, Vejle, Denmark) breast board with head holder and both arms lifted above the head. All plans were created for Millennium 120 multileaf collimator (MLC) and Varian Clinac iX linear accelerator (Varian Medical systems, USA). Due to Aria version update during the study, either PRO version v11.0.31 or PO v13.6.23 was used for optimization. The dose was calculated with 0.25‐cm grid size using Analytic Anisotropic Algorithm (AAA) either v11.0.31 or v13.6.23, using only one version for a given patient.

For all patients, the VMAT plans were designed as described by Boman et al.^6^ Thus, the VMAT plans consisted of four partial arcs or, in case of bilateral treatment, of eight partial arcs.^6^ Based on the beam's eye view images, the arcs were designed to avoid the lung, the heart, and the contralateral breast. The collimator angles (CA) in left‐sided plans were between 10° and 30° for the anterior partial arcs, and complement angles were used for the lateral partial arcs.^6^ For right‐sided plans, the opposite angles were used. The field size was restricted to 15–18 cm in the left‐right direction, that is, direction of MLC movement, to allow for optimal MLC modulation. As a modification to the split‐arc design described in literature,^6^ in some cases a small gap of 10°–20° between the frontal and lateral subarcs was used. This aided in minimizing the heart dose in right‐sided cases. A virtual water‐equivalent bolus of 5 mm was used in the areas where the PTV extended to the skin, but the final dose was calculated without the bolus, using normalization of mean dose of 100% to the PTV cropped 5 mm inside from the body contour (PTV‐5 mm).

All patients selected to this study had traditional half‐blocked FinF plans, which were used as a reference in dosimetric analyses. The FinF plans consisted of two tangential half‐blocked fields for the breast or chest wall, and three fields for the supraclavicular lymph‐node region, including two anterior and one posterior field.^11^ The tangential fields were extended laterally to the air by 2.5 cm to create the skin flash.

### 2.C kV and CBCT imaging

Tattoo‐based patient setup and 2D kV image guidance was used for all patients.^11^, ^12^, ^13^ At least daily tangential and weekly orthogonal kV were imaged for the DIBH patients, and weekly tangential kV for the free breathing patients.^11^, ^12^, ^13^ The tolerances for chest wall were 5 mm in the cranio‐caudal (C‐C) and 4 mm in the combined lateral and anterior–posterior (A‐P) directions.

**Figure 1:**
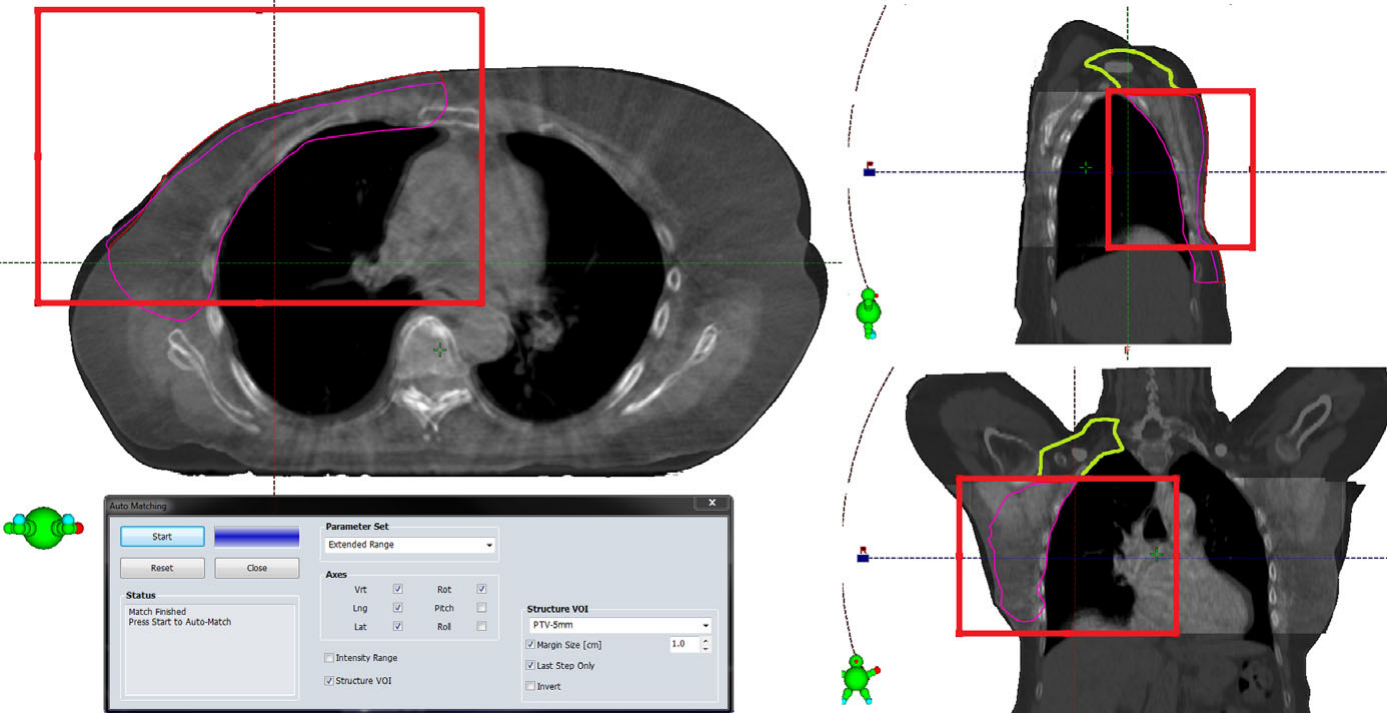
Region of interest for matching the CBCT image to the CT image is drawn with the red rectangle. The matching region of interest is centered to the PTV (red contour), effectively excluding the spine. The division of PTV‐5 mm into PTVb/c (magneta contour) and PTVsclav (green contour) is made on the level where the original PTV (red contour) reaches the skin, shown in the sagittal and coronal views.

Weekly CBCT was acquired in order to analyze the dose in the possibly swollen or deformed breast or chest wall. The CBCTs were registered to the original planning CT using automatic registration in the Eclipse software. The registration volume was restricted to the PTV and chest wall, largely excluding the spine (Fig. 1). To study the dosimetric effect of the soft tissue deformations and posture changes seen in the CBCT images while keeping the dose calculations similar to the original plan, all comparative dose calculations were performed by modifying the original CT with Boolean operators as follows:

1. The CBCT‐based body contour was used in creating outside and inside structures with Boolean subtraction operators, where the BODY contour in the CBCT was extending outside or inside of the BODY contour of the original CT, respectively.
2. The outside structure consisted of the swollen tissue and was assigned to −100 HU as in fat tissue.
3. The inside structure consisted of areas of tissue shrinkage and was assigned to −1000 HU as in the air.
4. The final modified body contour was created as (BODY_CT OR BODY_CBCT) SUBTRACT BODY_shrinkage.

The actual treatment was based on online 2D kV‐match, and the isocenter error was evaluated post‐treatment in the CBCT images using automatic registration. The original plan was recalculated with the surface‐corrected structure set using the monitor units (MUs) of the original plan. Three different dose calculations were performed:

- the isocenter positioned with the 3D match of the automatic offline CBCT registration, with table rotation correction included in the match (3D + rot)
- the isocenter positioned with the online 2D kV image match including the same couch rotation as in the 3D + rot match (2D + rot)
- the same online 2D‐matched isocenter as in (ii) without couch rotation correction (2D).

The tolerances for chest wall and heart were 5 mm in C‐C and 4 mm in lateral and A‐P directions.^12^, ^13^ The three dose distributions based on different matching techniques (3D + rot, 2D + rot, 2D) were calculated for both the VMAT and FinF plans, resulting in six dose distributions for each CBCT image.

### 2.D Image analysis

For each surface‐corrected structure set, the heart and humeral head(s) were modified from the planning CT to match the CBCT image. Additionally, the PTV border was modified to match the skin surface seen in the CBCT image. An experienced oncologist reviewed all modifications. For nine patients the humeral head was not shown in the CBCT due to the limited image field of view (FOV) of 16 cm in the C‐C direction, and only 16 patients were included in the analyses of humeral head. Also the PTV and heart were partially cut out due to the restricted FOV in CBCT, but their position was evaluated based on the part shown on CBCT. In order to minimize the uncertainties caused by the cranially missing CBCT data, PTV‐5 mm was evaluated also by dividing it into the breast or chest wall (PTVb/c) and the supraclavicular (PTVsclav) regions. The junction between PTVb/c and PTVsclav regions was made on the CT slice where the original PTV was no longer extended to the skin (Fig. 1). The PTVb/c was therefore mostly unaffected by missing CBCT data and contained the errors resulted from the tissue deformations and isocenter positioning. Instead, the results of PTVsclav included only errors due to isocenter positioning, and the anatomical deformations were not included.

The PTV dose minima were evaluated using V95% and V90%, and dose maxima using V105% and D2 cc. Dose conformity index (CI) and homogeneity index (HI) were evaluated for the PTV in each calculation. The Paddick CI,

(1) $$\text{CI}=\frac{V95(\text{PTV})(\text{cc})}{V(\text{PTV})(\text{cc})}\frac{V95(\text{PTV})(\text{cc})}{V95(\text{PTV})(\text{cc})}$$

was used, where V95(PTV) (cc) was the volume which receives at least 95% of the prescribed dose in the PTV, V(PTV)(cc)was the total volume of PTV, and V95(cc) was the volume of the whole body which received at least 95% of the prescribed dose. For the HI,

(2) $$\text{HI}=\frac{D2\%(\text{PTV})-D98\%(\text{PTV})}{D50(\text{PTV})}$$

DX%(PTV) (Gy) indicated the dose received by X% of the volume of PTV, and D50(PTV) (Gy) was the median dose of the PTV. For CI, the value one means the highest conformity possible, and lower values mean decreased conformity. For HI, lower values mean better homogeneity.

Dose to the heart was evaluated as mean dose Dmean(heart) (Gy), and as percentages of volume receiving the doses of 20, 10, and 5 Gy (V20(heart) (%), V10(heart) (%), and V5(heart) (%), respectively); and D2cc(heart) (Gy) as the maximum dose received by the volume of 2 cm^3^. Similarly, for the humeral head V15(humer) (%) and D2cc(humer) (Gy) were evaluated for 16 patients with the humeral head visible in the CBCT. The lungs were not evaluated due to partial CBCT coverage and the uncertainties in the HU values in the modified anatomy.

**Figure 2:**
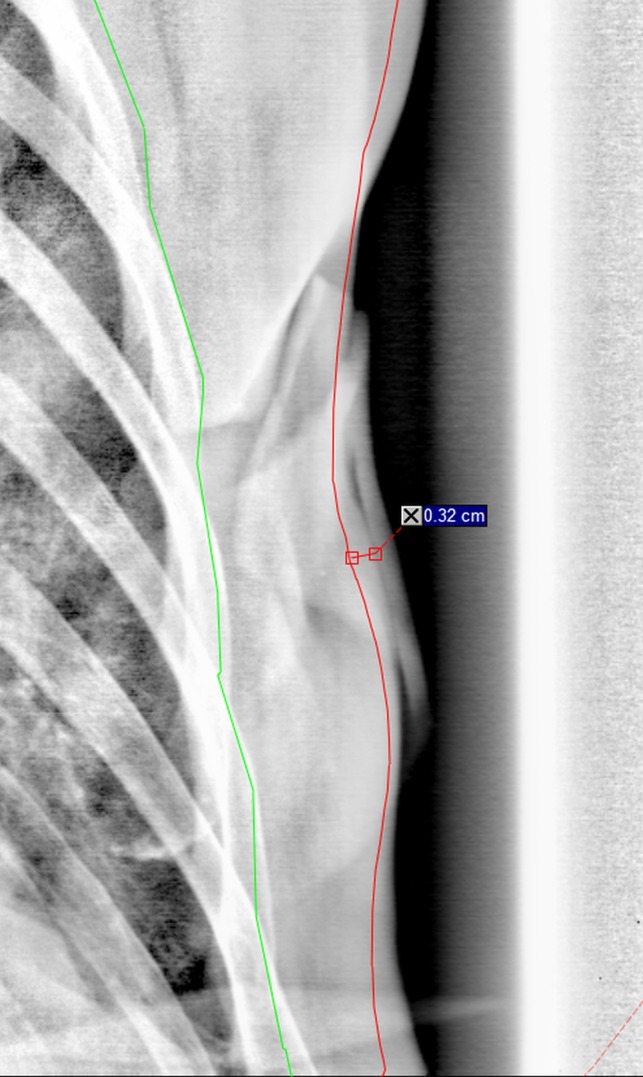
Measurement of tissue swelling or shrinkage d_s_ in the tangential image as the distance of the skin from the original CT‐based skin contour.

In addition to the CBCT analyses, the amount of breast tissue swelling or shrinkage (d_s_ (mm), minus sign indicating the shrinkage) was measured by a maximum skin surface change seen in the tangential kV‐images when compared to the skin surface outline seen in the planning CT image as demonstrated in Fig. 2. Residual errors of the 2D online match were evaluated for the vertebrae, sternum, and ribs.^12^ For the DIBH patients, the residual error in breath hold level (BHL) was measured on the CBCT images in both A‐P and C‐C directions using the difference between two independent CT‐CBCT registrations: first matching the vertebra, and then matching the sternum.^14^

### 2.E. Statistical analyses

Statistical analyses were computed using SPSS (v22, IBM corp., New York, NY, USA). Normality of data distribution was evaluated with the Kolmogorov–Smirnov test with significance level of *P* < 0.05. The Paired Samples *t*‐test was used for normally distributed data, and the Wilcoxon Signed Ranks Test for non‐normally distributed data. Bonferroni correction was used to correct for multiple analyses. Statistical significance was considered when *P* < 0.05. Correlation was tested with the Pearson correlation coefficient or Spearman's rho test for normal and non‐normal distributions, respectively.

## 3 RESULTS

For every patient, three to six CBCT images were obtained, except for one large‐sized bilateral case (Pat #9), for whom the left and right sides were imaged separately. For the 24 patients, 149 CBCT images were acquired and analyzed for this study resulting total of 894 dose calculations.

### 3.A Original plan quality

**Figure 3:**
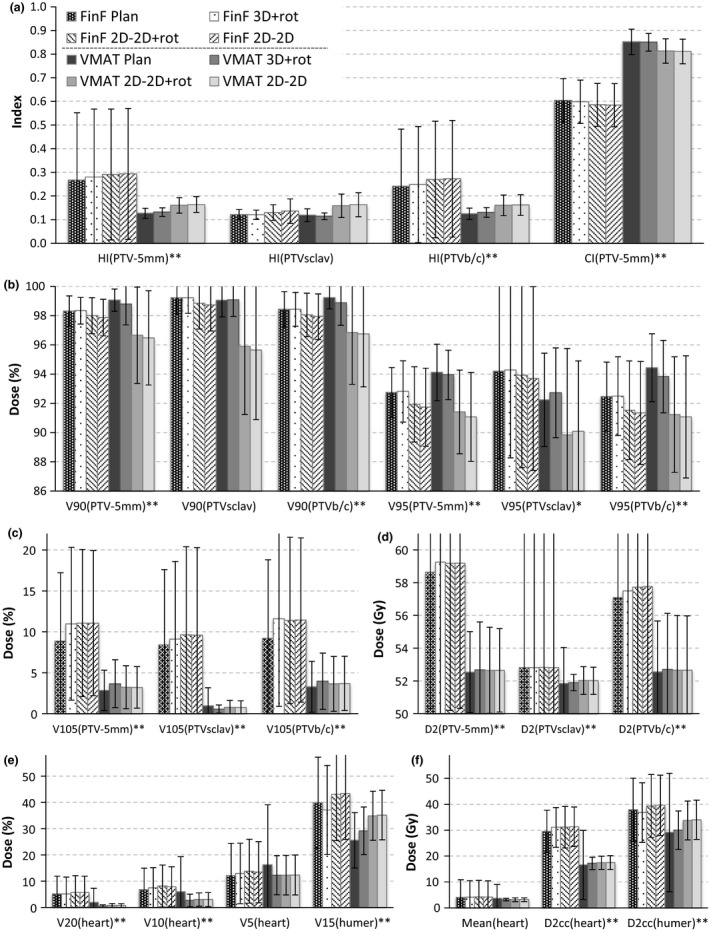
The DVH parameters for FinF (patterned bars) and VMAT (solid‐colored bars) original plans and different setup methods (3D‐3D + rot, 2D‐2D + rot, and 2D‐2D) based on modified patient geometry in the CBCT images. The averages of all patients are presented with standard deviations. *P < 0.05; **P < 0.01 for the paired test between the *original* FinF and VMAT plans. Other differences (based on CBCT‐based geometry) are reported in Tables 3, 4, and 5.

In the original plans, the HI and CI were better in the VMAT plans than in the FinF plans (Fig. 3). For the CI, the differences were more pronounced. The differences in other DVH parameters of the PTV were higher V90%(PTV) and V95%(PTV) except for the clavicular region; and lower V105% and D2 cc in the VMAT than in the FinF plans (Fig. 3). The D2% of the heart was lower in the VMAT than in the FinF plans. Otherwise the heart doses were closely similar, with large doses going to a slightly larger volume in the FinF plans, and with low doses spreading to a slightly larger volume in the VMAT plans. For the humeral head, the VMAT doses were significantly lower than the FinF doses.

### 3.B Tissue deformation

The range of tissue deformations d_s_ in the tangential images (Fig. 2) was from −5 to 27 mm, with median swelling of 2 mm and interquartile range from 0 to 4 mm. No correlation was found between increasing swelling and time from the first fraction (Spearman's rho = −0.109). Instead, some patients had swelling in the beginning, some at the end of the treatment course, and several had no or only minor swelling, shrinkage, or deformation. The correlations between skin deformations d_s_ (Fig. 2) and dose parameters for V95%, V105%, and D2 cc for target volumes (PTV‐5 mm, PTVsclav, PTVb/s) are presented in Table 2. The effect of skin deformations on dose minima (V90% and V95%) was limited, but the dose maxima (V105% and D2 cc) were slightly affected; increasing the maxima with larger swelling in the FinF plans; and decreasing the maxima in the VMAT plans.

**Table 2:** Correlation coefficients for correlations between the tissue deformations d_s_ (Fig. 2) measured in tangential images and DVH changes in PTV dose minima (V90% and V95%) and maxima (V105% and D2 cc). Values are bolded where *P* < 0.05. Spearman's rho test

	V90%			V95%			V105%			D2 cc		
	PTV‐5 mm	PTV sclav	PTVb/c	PTV‐5 mm	PTV sclav	PTVb/c	PTV‐5 mm	PTV sclav	PTVb/c	PTV‐5 mm	PTV sclav	PTVb/c
FinF												
3D + rot	–	–0.122	–0.070	0.080	0.062	0.050	0.115	0.133	0.099	0.111	**0.198**	–0.048
2D + rot	–0.026	–0.011	–0.088	0.052	0.175	–0.020	0.165	0.193	**0.187**	0.138	**0.244**	0.057
2D	–0.067	0.052	–0.092	0.003	0.178	–0.025	0.166	0.171	**0.180**	**0.190**	**0.245**	0.100
VMAT												
3D + r ot	–0.172	0.136	–0.184	–0.205	–0.033	–0.292	–0.137	–0.070	–0.189	–0.140	–0.054	–0.173
2D + r ot	–0.094	0.092	–0.050	–0.076	–0.142	–0.069	–0.290	–0.254	–0.266	–0.261	–0.293	–0.233
2D	–0.153	0.085	–0.087	–0.086	–0.130	–0.070	–0.311	–0.183	–0.305	–0.285	–0.268	–0.261

Five of the studied cases were replanned during the treatment. In one unilateral case (Pat #17), essential seroma‐related soft tissue swelling d_s_ of maximally 27 mm led to a clinically significant decrease in V90%(PTVb/c) dose coverage. This swelling required new planning CT and replanning of the treatment. Two bilateral cases (Pat #8 and #23) were replanned due to deformations exceeding 10 mm related to seroma cavity (d_s_ 15 and 12 mm, respectively). In all three cases, also clinically significant decrease was seen in the dose coverage in V90%(PTVb/c) with the 2D match, but in the two bilateral cases 3D + rot match would have decreased the influence of swelling, improving the V90%(PTVb/c) from 91.4% to 99.9% and from 92.8% to 99.6% for Pat #8 and #23, respectively. One patient with SIB (Pat #15) was replanned due to setup difficulties to ensure the dose coverage in the boost volume. In this case, no new CT was needed but the booster margins were enlarged from 4 to 6 mm. One case (Pat #12) was replanned due to a very large breast with a consistent postural change in the direction of tangential image field. In this case, a small tangential change (d_s_ = −4 mm) caused V95%(PTVb/c) to drop from 99.8% to 92.8%. The postural change was seen already at the first CBCT imaged at the second fraction, and the consistency was verified with the second CBCT. In this case, the 3D + rot match would have improved the V90%(PTVb/c) compared to the 2D match from 92.8% to 99.2%.

The majority of the cases were not replanned (19/24). For them the 2D match‐based V90%(PTVb/c) was on average 97.7%. For comparison V90%(PTVb/c) was on average 93.7% for the five replanned cases prior to the replanning.

### 3.C Isocenter error

For all FinF and VMAT recalculated plans (3D + rot, 2D + rot and 2D) the HI and CI indicated reduced plan quality when compared to the original plans. Differences between the original and recalculated plans indicated consistently that the 3D + rot match was closest to the original plan, for 2D + rot and 2D match the differences were slightly larger for both the VMAT and the FinF plans [Fig. 3(a)]. The changes in HI and CI from the original plan to the recalculated plans were statistically significant (*P* < 0.05) in nearly all comparisons [Fig. 3(a)]. Only the change in HI in the FinF plans was not significant for the 3D + rot match for PTV‐5 mm and PTVb/c; and for the 2D + rot setup for PTVsclav. The differences between the three matching schemes were statistically significant except for the HI of both the supraclavicular and breast/chest wall regions between the 2D + rot and 2D in the VMAT plans.

The differences between the original plan and reconstructions after different matching methods for V90%(%), V95%(%), V105%(%), and D2 cc(Gy) for PTV‐5 mm, PTVsclav and PTVb/c are presented in Figs. 3(b)–3(d). The differences reflect the trend of the HI and CI, with decreasing PTV coverage and increasing dose maxima (V105%, D2 cc) induced by the anatomic deformations and isocenter errors. The maximum dose D2 cc increased when patient setup inaccuracies were introduced, but it was not dependent on the matching method. The individual *P*‐values for the differences between plan and different matching methods are presented in Table 3 for the dose minima and Table 4 for the dose maxima.

**Table 3:** *P*‐values for differences in the dose minima V90%(%) and V95%(%). Pairwise comparisons are performed first as differences from the original plan to actual CBCT‐based patient geometry using each of the three matching techniques, and second between the three matching techniques. Statistically significant values are in bold

	V90%			V95%		
	PTV‐5 mm	PTVsclav	PTVb/c	PTV‐5 mm	PTVsclav	PTVb/c
FinF						
Plan vs. 3D + rot	**0.000**	0.823	1.000	0.641	0.812	1.000
Plan vs. 2D + rot	1.000	0.122	**0.003**	**0.001**	1.000	**0.005**
Plan vs. 2D	1.000	**0.041**	**0.001**	**0.000**	1.000	**0.000**
VMAT						
Plan vs. 3D + rot	**0.002**	**0.000**	**0.000**	**0.000**	**0.000**	**0.000**
Plan vs. 2D + rot	**0.026**	**0.000**	**0.000**	**0.000**	**0.000**	**0.000**
Plan vs. 2D	**0.011**	**0.000**	**0.000**	**0.000**	**0.000**	**0.000**
FinF						
3D + rot vs. 2D + rot	**0.013**	**0.025**	**0.008**	**0.000**	1.000	**0.000**
3D + rot vs. 2D	**0.000**	**0.004**	**0.002**	**0.000**	0.324	**0.000**
2D + rot vs. 2D	**0.000**	1.000	0.076	**0.023**	0.706	0.860
VMAT						
3D + rot vs. 2D + rot	**0.000**	**0.000**	**0.000**	**0.000**	**0.000**	**0.000**
3D + rot vs. 2D	**0.000**	**0.000**	**0.000**	**0.000**	**0.000**	**0.000**
2D + rot vs. 2D	**0.000**	0.420	0.480	**0.000**	0.114	0.370

**Table 4:** *P*‐values for differences in the dose maxima V105%(%) and D2 cc(Gy). Pairwise comparisons are performed both as differences from the original plan to the three matching techniques, and as differences between the matching techniques. Statistically significant values are in bold

	V105%			D2 cc		
	PTV‐5 mm	PTVsclav	PTVb/c	PTV‐5 mm	PTVsclav	PTVb/c
FinF						
Plan vs. 3D + rot	**0.000**	0.060	**0.000**	**0.003**	0.175	0.330
Plan vs. 2D + rot	**0.000**	0.188	**0.005**	**0.001**	0.399	0.294
Plan vs. 2D	**0.000**	0.399	**0.003**	**0.001**	0.663	0.077
VMAT						
Plan vs. 3D + rot	**0.000**	**0.000**	**0.000**	**0.000**	**0.000**	**0.000**
Plan vs. 2D + rot	**0.040**	**0.000**	0.997	0.032*	**0.000**	0.978
Plan vs. 2D	0.053	**0.000**	0.837	0.038*	**0.000**	1.000
FinF						
3D + rot vs. 2D + rot	1.000	1.000	1.000	1.000	1.000	1.000
3D + rot vs. 2D	1.000	1.000	1.000	0.978	1.000	1.000
2D + rot vs. 2D	1.000	1.000	1.000	1.000	1.000	0.279
VMAT						
3D + rot vs. 2D + rot	1.000	0.207	0.927	1.000	0.372	0.538
3D + rot vs. 2D	1.000	0.413	1.000	1.000	0.402	0.770
2D + rot vs. 2D	1.000	1.000	1.000	1.000	1.000	1.000

**Figure 4:**
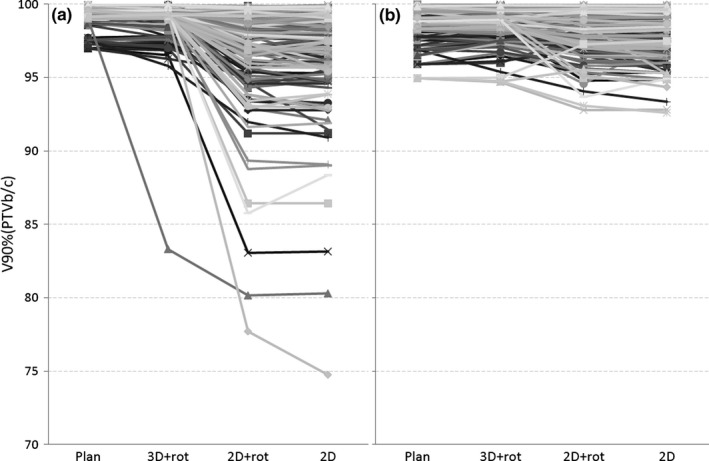
The decrease in V90%(PTVb/c) from the plan to treatment using different patient setups (3D + rot, 2D + rot, and 2D) for the VMAT (a) and FinF (b) techniques. Each line represents an individual fraction.

The change in V90%(PTVb/c) from the original plan to the actual CBCT‐based patient geometry using different matching methods is shown for individual fractions in Fig. 4 for VMAT (A) and FinF (B). Some patients had individual fractions where the 2D online‐matched isocenter error was considerable compared to the CBCT match, but at the previous and next fraction the error was small. These random isocenter errors had no correlation with time from the first fraction for neither all patients combined (*P* > 0.25), nor as individual patients. Also, no correlation between the isocenter error and the skin deformation d_s_ was found (*P* > 0.33). In VMAT treatments, in terms of PTV coverage of V90% the dose decrease in the 2D online match would have been corrected if 3D + rot match had been used for all patients and fractions (Fig. 4), except for one fraction of Pat #17 with the 2.7‐mm seroma. In the FinF plans the V90% difference between 2D and 3D + rot match was small (Fig. 4). If the 2D match was used, the FinF plans provided better PTV coverage (*P* < 0.001). When the 3D + rot match was used, VMAT proved slightly better in PTV coverage (*P* < 0.001). The difference between 2D and 2D + rot was negligible.

The correlations between the dose parameters of the PTV coverage and isocenter errors in the online 2D match in the A‐P, C‐C, and lateral direction, and in the patient rotation were modest at maximum for both FinF and VMAT recalculated plans. The HI(PTV‐5 mm) was modestly correlated with the A‐P isocenter error (Spearman's rho = 0.510) in the VMAT plans. This was accompanied by weak correlations (ρ = 0.330–0.433) in V95%, V105%, and D2 cc. In the FinF plans, the individual parameters were weakly correlated with the A‐P isocenter error (ρ < 0.5), but neither HI nor CI were affected by these.

### 3.D Heart and humeral head

The doses to the heart and humeral head are shown in Fig. 3(e)–3(f), and the *P*‐values in Table 5. The V20%, V10%, V5% and the mean dose to the heart increased slightly in the FinF recalculated plans (3D + rot, 2D + rot and 2D) but decreased in the VMAT recalculated plans when compared to the original plans. The maximum dose (D2cc_HEART_) increased slightly in the recalculated plans when compared to the original plans using both FinF and VMAT technique. The heart dose change (from the planned to the actual 2D match based) correlated weakly with the A‐P isocenter error both in the FinF plans (*P* < 0.05, Spearman's rho = 0.201…0.248) and in the VMAT plans (*P* < 0.05, *r* = 0.393…0.547).

**Table 5:** *P*‐values for differences in the heart and humeral head doses. Pairwise comparisons from plan to actual CBCT‐based dose in the modified patient geometry, and pairwise comparisons between the different matching techniques. Statistically significant values are in bold

	Heart					Humeral head	
	V20 Gy	V10 Gy	V5 Gy	D2 cc	Mean	V15 Gy	D2 cc
FinF							
Plan vs. 3D + rot	0.828	0.088	0.366	**0.003**	0.557	**0.000**	**0.000**
Plan vs. 2D + rot	1.000	0.672	0.601	1.000	1.000	0.023	0.225
Plan vs. 2D	1.000	0.820	1.000	0.176	1.000	0.023	0.238
VMAT							
Plan vs. 3D + rot	1.000	**0.007**	**0.030**	**0.004**	0.056	0.180	**0.001**
Plan vs. 2D + rot	0.450	**0.022**	0.394	0.075	1.000	0.194	1.000
Plan vs. 2D	0.305	**0.015**	0.213	0.057	0.859	1.000	1.000
FinF							
3D + rot vs. 2D + rot	1.000	1.000	1.000	0.238	1.000	**0.000**	**0.000**
3D + rot vs. 2D	1.000	1.000	1.000	1.000	1.000	**0.000**	**0.000**
2D + rot vs. 2D	1.000	1.000	1.000	1.000	1.000	1.000	0.537
VMAT							
3D + rot vs. 2D + rot	1.000	1.000	1.000	1.000	0.708	**0.000**	**0.000**
3D + rot vs. 2D	1.000	1.000	1.000	1.000	1.000	**0.000**	**0.000**
2D + rot vs. 2D	0.457	1.000	1.000	1.000	**0.006**	1.000	1.000

For the residual errors of bony landmarks after the 2D match, the C‐C residual error of the spine in the A‐P image was weakly correlated with the heart dose change in both FinF (*P* < 0.05, *r* = 0.230…0.443) and VMAT (*P* < 0.002, *r* = 0.333…0.463) plans. The lateral and A‐P residual errors of the vertebrae, sternum, and ribs in the 2D match were not significantly correlated with the heart dose change, and nor were the residual errors of ribs in the tangential 2D images.

In the DIBH patients, small to moderate changes in the BHL were seen in the A‐P direction on the CBCT images (median 0 cm, range −0.8…0.8 cm, interquartile range −0.3…+0.2 cm). These changes correlated modestly with all heart dose parameters in the 2D match‐based FinF treatment (Spearman's rho = 0.278…0.410) and VMAT treatment (ρ = 0.381…0.424). In the C‐C direction a similar but slightly weaker correlation was seen in both FinF (ρ = 0.257…0.366) and VMAT (ρ = 0.124…0.385) technique.

The changes in the humeral head were only computed for the portion of patients (16 patients, 66 CBCT images) with at least part of the humeral head visible in the CBCT. The dose changes to the humeral head varied largely in both directions, depending on the accuracy of the shoulder position.

## 4 DISCUSSION

The radiotherapy of breast cancer is still often accomplished using 3D conformal technique, such as the half‐blocked tangential FinF method. However, there is an increasing trend toward intensity‐modulated radiation therapy (IMRT) techniques and VMAT.^3^, ^4^, ^5^, ^6^, ^7^, ^8^, ^9^ In FinF and IMRT, there is a possibility to extend the field to the air outside the body to account for eventual changes of shape or swell of the breast tissue, but in Varian's Eclipse VMAT planning system the possibilities are limited to the use of an optimization bolus. Our clinic has limited the thickness of the bolus to 5 mm in order to avoid large dose maxima in the final dose calculation which is performed without the bolus. Therefore, the knowledge of actual changes in the breast or chest wall tissue is needed in order to properly cover the PTV during the RT treatment course.

### 4.A Tissue deformations

Mancosu et al.^7^ studied small deformations of around 3–4 mm for DIBH VMAT treatments and found that the change in V95%(PTV) was less than 3.5%. Kusters et al.^8^ studied the chest wall movement in A‐P direction in a simulation for VMAT DIBH treatments and found that the CTV coverage was still acceptable if the errors seen were smaller than 7.5 mm in either direction. Our results suggested that V90%(PTV‐5 mm) was acceptable and no replanning was required for VMAT plans for most of the patients (19/24 = 79%) if the soft tissue deformation was small and the patient setup and image match was successful.

The most demanding cases were patients with seroma that required frequent punctuation. In such cases, the advantage of VMAT treatment over FinF may be questionable, especially if 2D match is used. One solution might be to generate several plans with different skin contours and to select a plan of the day that corresponds to the skin contour. Alternatively, with the CBCT match moderate seroma cases can be safely treated. Indeed, even two bilateral cases with seroma of larger than 10 mm were properly covered by 90% dose. With the CBCT match, only Pat #17 with 2.7‐cm swell in the tangential image would have been clinically unacceptable (V90%(PTVb/c) = 83%).

### 4.B Isocenter errors

Patient inter‐ and intrafraction motion may be in the range of several millimeters, including isocenter shifts, rotations, and errors in breath hold level. These inaccuracies may become important if imaging is insufficient.^11^, ^15^ In our study, only minor decrease in the PTV coverage was present when moving from the original plan to the automatic CBCT match with tattoo‐based setup and anatomical deformations. However, the plan quality decreased considerably when an isocenter error was present in the 2D + rot match; and slightly more if the residual error in the patient rotation was not corrected with the couch rotation in the 2D match. The changes to the PTV coverage were small but consistent.

Outliers with large dose changes were seen where the online match had failed on an individual day, although the neighboring fractions were well matched. Individual outliers may be expected in a busy clinic with RTTs having varying amount of experience. Furthermore, outlier fractions may occur if imaging is not performed on every fraction. These will deteriorate the dose distribution of a VMAT treatment more than a traditional treatment. The decreasing PTV coverage in the breast or chest wall region caused by isocenter errors was more pronounced in the VMAT plans than in the FinF plans due to the large amount of modulation in the VMAT plans. For these reasons an automatic CBCT match with careful match review is recommended over online 2D match prior to VMAT treatments.

### 4.C Heart dose

A small isocenter error in the A‐P direction correlated with the heart dose change. This was expected as the high‐dose volume got closer to or further from the heart. In addition, correlation was found between the heart dose and the C‐C residual error of the spine. This was likely due to the effect of C‐C breathing motion. If the A‐P BHL is lower than planned, it is more likely that positional errors also occur in the C‐C direction.^14^ Thus, if in 2D image guidance the match of lateral image is performed with a compromise between the sternum and spine, but prioritizing the sternum, then the C‐C residual error of the spine is likely connected with the altered breathing motion. Indeed, we have seen several patients whose breathing movement is equally or even more pronounced in the C‐C than in the A‐P direction. This finding was supported by the residual error in the BHL of DIBH patients, where both the A‐P and C‐C error affected the heart dose. Therefore, attention should be paid not only on the A‐P but also on the C‐C breathing motion. Errors in the BHL should be corrected by adjusting the BHL limits and/or instructing the patient based on visual assessment of the lateral image.

### 4.D Supraclavicular region

The accuracy of the supraclavicular region (PTVsclav) was limited by the CBCT image size and should only be considered for isocenter positioning errors, not anatomical deformations. The PTV contours could not be verified outside the CBCT image range. Due to the optimization criteria, the irradiation of the supraclavicular regions traverses primarily through the A‐P directions in VMAT treatments. However, unlike in traditional planning, part of the irradiation enters the patient from the lateral direction. The inclusion of lateral beams increases the uncertainty of planned dose delivery if the shoulder moves especially in the C‐C or A‐P direction.^16^

### 4.E Imaging protocols

Using Varian OBI low‐dose thorax CBCT mode, the CBCT‐induced dose to the breast is in the range of 0.4–1 cGy per fraction.^17^, ^18^, ^19^ The dose to the heart is in the range of 0.4–1 cGy, and to the lung 0.5–0.7 cGy.^17^, ^18^, ^20^ Even though these are not large doses, the cumulative dose of daily CBCT does increase the doses to the OARs compared to 2D kV imaging. However, this might be compensated by the decreased heart doses with the more accurate setup. Furthermore, the doses are nearly halved in Varian TrueBeam CBCT.^20^ The modification of the imaging protocols will decrease the doses also in the OBI version; down to 0.20, 0.20, 0.27, 0.13, and 0.16 cGy in the ipsi‐ and contralateral lung, heart, ipsi‐, and contralateral breast, respectively, compared to the respective doses of a kV‐kV pair: 0.19, 0.00, 0.16, 0.42, and 0.05 cGy.^18^

Unfortunately, tangential 2D imaging is sensitive for the gantry angle in detecting local swelling or deformation, as their location and direction may vary. On the other hand, CBCT image size is limited for most onboard imagers and does not suffice in the simultaneous accurate setup of the supraclavicular region and the breast/chest wall. For the tangential FinF plans online 2D match was sufficient to ensure proper PTV coverage in spite of relatively large isocenter position errors detected in the offline CBCT match. Instead, for the VMAT plans the 3D + rot match was seen superior. The CBCT match is therefore recommended to be used as frequently as possible (e.g., daily) for patient positioning in VMAT treatments both to decrease the average error, and to eliminate large random errors.

## 5 CONCLUSION

Considering the soft tissue deformations and breast tissue swelling during the course of radiotherapy of the breast, the actual dosimetric parameters would be similar to the plan for both VMAT and FinF plans if 3D image matching was used. Especially for VMAT plans, the changes in the dosimetric parameters became worse with the online 2D matching methods. In VMAT treatments, a daily automatic CBCT matching along with monitoring of the skin surface is recommended. Large deformations in any direction should be evaluated in terms of their clinical relevance, and the potential need for replanning should be investigated.

## CONFLICT OF INTEREST

The authors declare no conflicts of interest.
